# The Added Value of Trabecular Bone Score in Evaluating Fracture Risk Among Polish Women Aged 40–76 Years

**DOI:** 10.3390/jcm15114185

**Published:** 2026-05-28

**Authors:** Bożena Jaszczyk, Anna Nowakowska-Płaza, Barbara Stypińska, Iwona Sudoł-Szopińska, Brygida Kwiatkowska, Piotr Głuszko, Jakub Brzeziński, Robert Olszewski

**Affiliations:** 1 Outpatient Department, National Institute of Geriatrics, Rheumatology and Rehabilitation, Spartańska 1 Street, 02-637 Warsaw, Poland; bozena.jaszczyk@spartanska.pl; 2 Department of Internal Medicine, Pneumonology, Allergology, Clinical Immunology and Rare Diseases, Military Institute of Medicine, National Research Institute in Warsaw, 01-045 Warszawa, Poland; anowakowskaplaza@gmail.com; 3 Department of Molecular Biology, National Institute of Geriatrics, Rheumatology and Rehabilitation, Spartańska 1 Street, 02-637 Warsaw, Poland; barbara.stypinska@spartanska.pl; 4 Department of Radiology, National Institute of Geriatrics, Rheumatology and Rehabilitation, 02-637 Warsaw, Poland; iwona.sudol-szopinska@spartanska.pl; 5 Early Arthritis Clinic, National Institute of Geriatrics, Rheumatology and Rehabilitation, 02-637 Warsaw, Poland; brygida.kwiatkowska@spartanska.pl; 6 Multidisciplinary Osteoporosis Forum, 02-637 Warsaw, Poland; zruj@mp.pl; 7 Department of Gerontology and Public Health, National Institute of Geriatrics, Rheumatology and Rehabilitation, Spartańska 1 Street, 02-637 Warsaw, Poland; robert.olszewski@spartanska.pl; 8 Department of Ultrasound, Institute of Fundamental Technological Research, Polish Academy of Sciences, Pawińskiego 5B Street, 02-106 Warsaw, Poland

**Keywords:** trabecular bone score, fracture risk, osteoporosis, dual-energy X-ray absorptiometry

## Abstract

**Objectives:** Bone mineral density (BMD) assessment, the gold standard for diagnosing osteoporosis, does not account for bone quality and fracture susceptibility. Trabecular bone score (TBS) adds value to traditional densitometry. No studies have been conducted in the Polish population to date to confirm the association between TBS and fracture occurrence. This study aimed to evaluate the TBS derived from lumbar spine (L1–L4) dual-energy X-ray absorptiometry (DXA) scans in Polish women aged 40–76 years, both with and without osteoporotic fractures. The relationship between TBS, fracture risk (assessed by FRAX and TBS-adjusted FRAX), and BMD at the lumbar spine, femoral neck, and total hip was investigated. **Methods:** A total of 933 Caucasian women (760 without fracture and 173 with fracture) who underwent DXA examinations (Hologic Discovery A) between 2022 and 2024 were included. Lumbar TBS, BMD, and clinical fracture risk factors were analyzed, excluding subjects with scan artefacts or extreme BMI. Group differences were assessed using *t*-tests and chi-square tests. Pearson correlation was used to evaluate associations between TBS, age, and BMI. Logistic regression models assessed TBS and BMD as fracture discrimination, and model performance was compared using the Akaike Information Criterion (AIC) and the area under the receiver operating characteristic (ROC) curve (AUC). **Results:** TBS values were significantly lower in the fracture group (*p* < 0.001). TBS demonstrated negative correlations with age (r ≈ −0.36) and BMI (r ≈ −0.14). Low TBS values (≤1.23) were associated with the highest fracture prevalence (28.8%) and a threefold increased risk compared to high TBS (odds ratio = 3.0). Each one standard-deviation decrease in BMD or TBS T-score increased fracture risk by 56–67% (both *p* < 0.001). Models combining TBS and BMD improved discrimination, as indicated by higher AUC and lower AIC, with TBS remaining an independent predictor. In subgroups with osteopenia or osteoporosis, TBS retained statistical significance. **Conclusions:** TBS combined with BMD effectively discriminates fracture risk in Polish women and offers superior diagnostic accuracy compared to BMD alone. Integrating TBS with BMD enhances fracture accuracy. Routine assessment of TBS may improve clinical management of osteoporosis. Prospective studies are needed to confirm its long-term predictive value.

## 1. Introduction

So far, in everyday clinical practice, we do not have a reliable diagnostic technique that thoroughly assesses the quality of bone tissue and its fracture susceptibility. The gold standard for diagnosing osteoporosis remains the assessment of bone mineral density (BMD, g/cm^2^) by dual-energy X-ray absorptiometry (DXA). The WHO provided reference BMD values obtained from DXA, defining osteoporosis as a BMD value more than 2.5 standard deviations (SD) below the mean for healthy young adults (T-score ≤ 2.5 SD) [1]. While DXA enables the calculation of bone density, it does not provide insight into bone structure. A tool measuring disturbances in the microarchitecture of bone tissue is the trabecular bone score (TBS), calculated using computer software from images of vertebral bodies obtained from lumbar spine DXA scans (L1–L4). This software assesses variations in pixel grey levels in DXA images of the lumbar vertebrae, providing an indirect representation of bone microarchitecture, such as trabecular number, separation, and connectivity density. Bone with a higher number of trabeculae correlates with a higher TBS value and greater strength, whereas bone with fewer trabeculae shows a lower TBS and poorer quality [2]. This calculation enables both prospective and retrospective analysis of studies stored in the densitometer’s database. Low BMD is an important, but not the only, risk factor for fractures. However, TBS does not define osteoporosis; it is recognised as an additional, independent risk factor for fractures, and is now included alongside BMD in the fracture risk calculation tool FRAX-plus [3,4,5]. It is worth emphasizing that degenerative changes and spinal calcifications do not affect TBS values [6].

Currently, the usefulness of TBS in predicting fracture risk and guiding therapeutic decisions is recommended by more than 30 international scientific societies, including the European Society for Clinical and Economic Aspects of Osteoporosis, Osteoarthritis and Musculoskeletal Diseases (ESCEO) and the International Osteoporosis Foundation (IOF), under the auspices of the WHO Collaborating Centre for Epidemiology of Musculoskeletal Health and Aging [7,8]. Despite these, no large-scale studies have been conducted to date to assess the correlation between osteoporotic fractures and TBS in the Polish population. Existing analyses concern small groups of patients with postmenopausal osteoporosis [9,10] or secondary causes of osteoporosis [11,12,13,14,15,16,17].

This study aims to evaluate the value of the trabecular bone score (TBS) measurements in lumbar spine (vertebrae L1–L4) in a population of women aged 40–76 years with and without osteoporotic fractures, and to correlate TBS values (TBS and TBS T-score) with fracture risk and DXA BMD values measured at three sites: lumbar spine L1–L4 (DXA L1–L4), femoral neck (DXA neck), and total hip (DXA total hip), in a female population. This study thus represents the first large-scale study of TBS among a large group of Polish women.

## 2. Materials and Methods

### 2.1. Study Design and Setting

DXA scans were performed for diagnostic purposes in patients presenting for outpatient visits who had not previously been diagnosed with or treated for osteoporosis. All measurements were performed in 2022–2024 at the National Institute of Geriatrics, Rheumatology and Rehabilitation, Department of Radiology, Warsaw, Poland.

### 2.2. DXA and TBS Assessment

Data obtained during routine DXA scans using a Hologic Discovery A densitometer (Hologic, Waltham, MA, USA) were analysed. Bone mineral density (BMD) (g/cm^2^) and T-score (SD) were measured at three anatomical sites: femoral neck, total hip, and lumbar vertebrae L1–L4. Additionally, the TBS value (numeric, without unit) was calculated. TBS was obtained through automated analysis of lumbar spine DXA measurements using the TBS Medimaps software, version 3.0.3.0. The DXA scans were performed for diagnostic purposes. The study was conducted in a group of women who had never been previously diagnosed with or treated for osteoporosis.

### 2.3. Exclusion Criteria and Quality Control

**Figure 1:**
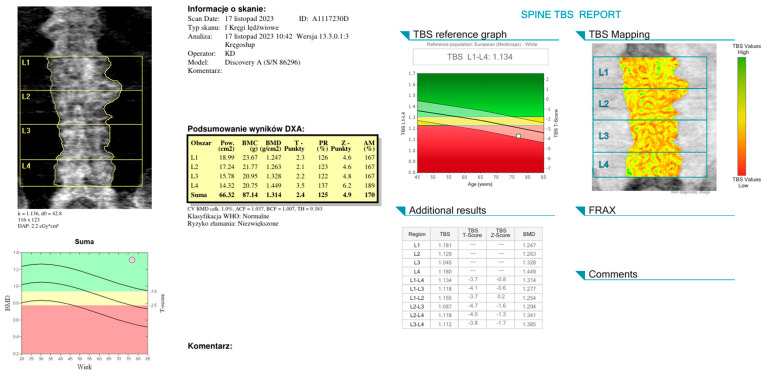
A sample of the lumbar spine BMD and TBS report. The DXA scan of the patient’s lumbar spine shows “normal” BMD values. Despite degenerative changes falsely increasing BMD, TBS indicates degraded bone microarchitecture.

DEXA reports that were unsuitable for analysis were excluded due to abnormalities within the examined region, such as lumbar vertebrae with compression fractures, haemangiomas, severe deformities, e.g., advanced rotoscoliosis, or imaging artefacts like metal elements of clothing such as buttons, zippers, as well as internal medical devices such as spinal stabilizers, vascular prostheses, vertebral cement augmentation, soft tissue calcifications, etc. Reports with patient positioning errors were excluded from our analysis. A sample of the lumbar spine BMD and TBS report is shown in Figure 1.

**Figure 2:**
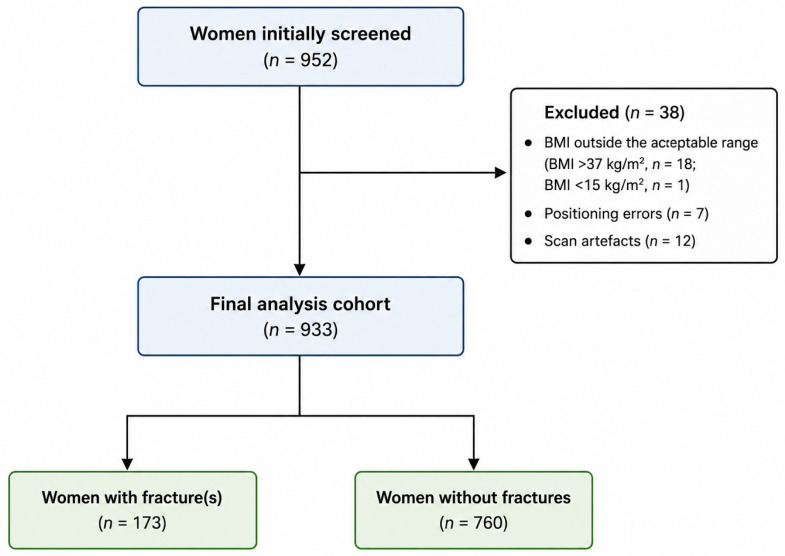
Patient selection and grouping flow diagram. Patient selection and grouping flow diagram. Of 952 women initially screened, 38 were excluded: 19 because of BMI outside the acceptable range (BMI > 37 kg/m^2^, *n* = 18; BMI < 15 kg/m^2^, *n* = 1), 7 due to positioning errors, and 12 due to scan artefacts. The final analysis included 933 women, who were subsequently divided into two groups: women with fracture(s) (*n* = 173) and women without fractures (*n* = 760).

In accordance with the official positions of the International Society for Clinical Densitometry (ISCD) [https://iscd.org/official-positions-2023, accessed on 19 January 2026], regarding densitometric study quality and the equipment manufacturer’s guidelines, TBS surveys for patients with BMI < 15 and >37 kg/m^2^ were excluded from the final analysis, as well. The algorithm is less technically reliable outside this range, due to extremely low or excessive soft-tissue mass and deterioration in DXA image quality (Figure 2) [8,18].

### 2.4. Ethics and Data Handling

Prior to data collection, approval was obtained from the Bioethics Committee at the National Institute of Geriatrics, Rheumatology and Rehabilitation (approval No. KBT-4/3/2025, 24 April 2025). For data collection and analysis, all patients’ personal data were encrypted and subsequently anonymized.

### 2.5. Clinical Data and Fracture Assessment

Patients’ medical records were analysed to obtain information on risk factors for osteoporosis and fractures, such as age, body mass index, comorbidities, smoking, alcohol consumption, and, most importantly, any previous fractures. Fractures were considered low-energy, based on a detailed medical history, clinical records, including X-ray studies, and consultants’ opinions. Fractures caused by high-energy trauma were excluded, and patients were classified as having no fragility fractures. In addition, patients with a loss of height <4 cm were referred for spinal X-rays. This procedure identified an additional 19 patients with asymptomatic (non-clinical) vertebral fractures. Fragility fractures were defined as those resulting from minor trauma (e.g., a fall from standing height) or spontaneous, and the localisation of the fracture referred to major osteoporotic fractures (MOFs), according to the definition of the National Institute for Health and Care Excellence (NICE): Clinical Guideline [19]. The adjudication of the fragility fractures group was performed by an investigator (a specialist in rheumatology with extensive experience).

Based on the above criteria, patients were divided into two groups:

1. With low-energy fracture(s).
2. Without a fragility fracture in medical history (Figure 2).

The Basic Fracture Risk Assessment Tool (FRAX) for the Polish population, as well as FRAX adjusted for TBS, were used to determine individual fracture risk [20]. Subsequently, based on the DXA measurements, patients were classified into one of three subgroups: with osteoporosis (T-score ≤ −2.5), with osteopenia (T-score < −1.0 and >−2.5), or with normal bone mass (T-score ≥ −1) [1]. Data from 933 Caucasian women aged 40–76 years were analysed.

Diagnostic concordance was assessed for the entire cohort based on DXA measurements of the femoral neck (n = 926) and lumbar spine (n = 921), as well as lumbar spine TBS (n = 634), and for subgroups stratified by age and BMI.

## 3. Statistical Analysis

All statistical analyses were performed using R software (R version 4.4.1, 14 June 2024 ucrt), “Race for Your Life,” Copyright (C) 2024, the R Foundation for Statistical Computing, Vienna, Austria. Data distribution was assessed visually using density plots and histograms. Distribution was also formally tested with the Shapiro–Wilk test. For normally distributed variables, results are presented as mean ± SD. For skewed variables, both mean ± SD and median (interquartile range) are reported. Differences in TBS and BMD variables between patients with and without fractures were tested using Welch’s *t*-test. Categorical comparisons, such as fracture prevalence across TBS categories, were evaluated with chi-square tests. Cochran–Armitage trend tests were used for ordered categories. Pearson correlation coefficients (r) were calculated to assess linear associations between TBS, age, and BMI. Univariable logistic regression was performed for each variable (TBS T-score, TBS, and BMD T-scores at various sites). Odds ratios (ORs) with 95% confidence intervals (CIs) were estimated. Multivariable logistic regression models were constructed, combining BMD T-scores and TBS, to assess independent predictive value. Model fit was evaluated using Akaike Information Criterion (AIC) and Bayesian Information Criterion (BIC). The predictive performance of each model was quantified using the area under the receiver operating characteristic curve (AUC). DeLong’s test for two correlated ROC curves was applied, to compare AUCs between models. Analyses were restricted to participants with available data for the included variables. The number of observations (N) is reported for each analysis. A two-sided *p*-value less than 0.05 was considered statistically significant.

## 4. Results

Patient characteristics are presented in Table 1.

The characteristics of the patients in the subgroups are presented in Tables S1–S3, which form part of the Supplementary Materials.

In our cohort, TBS-adjusted FRAX estimates were consistently higher than unadjusted FRAX-BMD values, both for major osteoporotic and hip fracture risk (Table 2).

**Figure 3:**
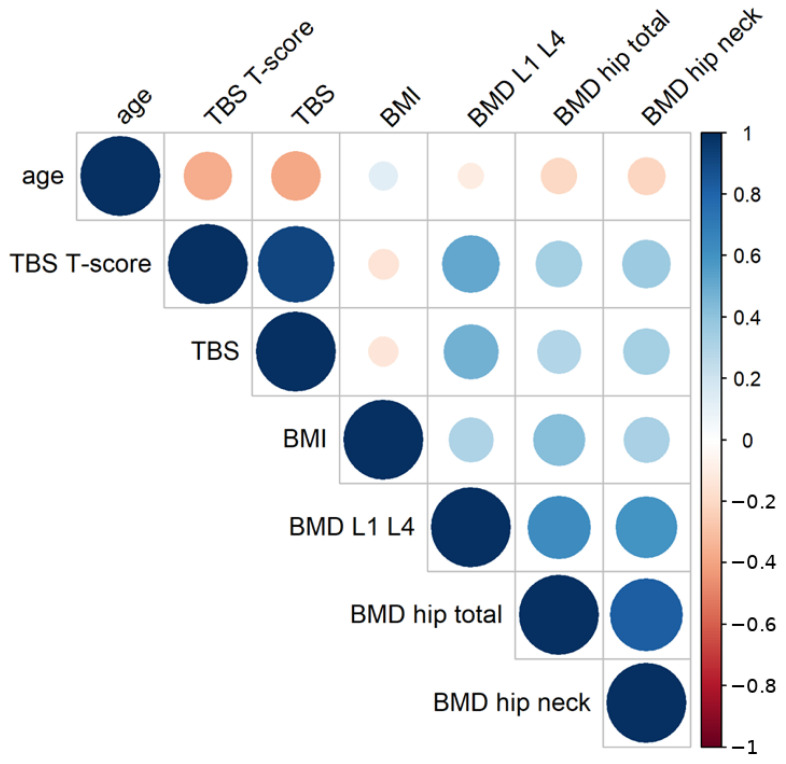
Correlation matrix of bone density parameters and clinical variables. The figure presents Pearson’s correlation coefficients between bone mineral density (BMD) values measured by DXA at the lumbar spine (L1–L4), total hip, and femoral neck, as well as the trabecular bone score (TBS), age, and body mass index (BMI). The colour intensity and size of the circles indicate the strength and direction of the correlations (blue for positive and red for negative correlations). Only statistically significant correlations are displayed (non-significant values are left blank). Hierarchical clustering (hclust) was applied to group variables with similar correlation patterns.

To evaluate the discriminative value of TBS, we compared women with and without osteoporotic fractures. The analysis demonstrated that both TBS and TBS T-scores were markedly lower in the fracture group, with differences reaching statistical significance (Welch’s *t*-test, *p* < 0.001 for both) (Table 1). TBS and TBS T-score were negatively correlated with both BMI and age. The correlations with age were stronger (r = −0.36 to −0.38, *p* < 0.001) than those with BMI (r = −0.14, *p* < 0.001), indicating that trabecular bone score decreases with advancing age and higher BMI (Table 3 and Figure 3). Although the age difference between groups reached statistical significance (59.82 ± 5.93 vs. 60.91 ± 5.64 years, *p* = 0.02), the absolute difference was small, and unlikely to be clinically meaningful. The positive predictive value (PPV) and negative predictive value (NPV) were additionally calculated for the optimal ROC cut-off, determined using the Youden index (Supplementary Materials Table S4).

**Figure 4:**
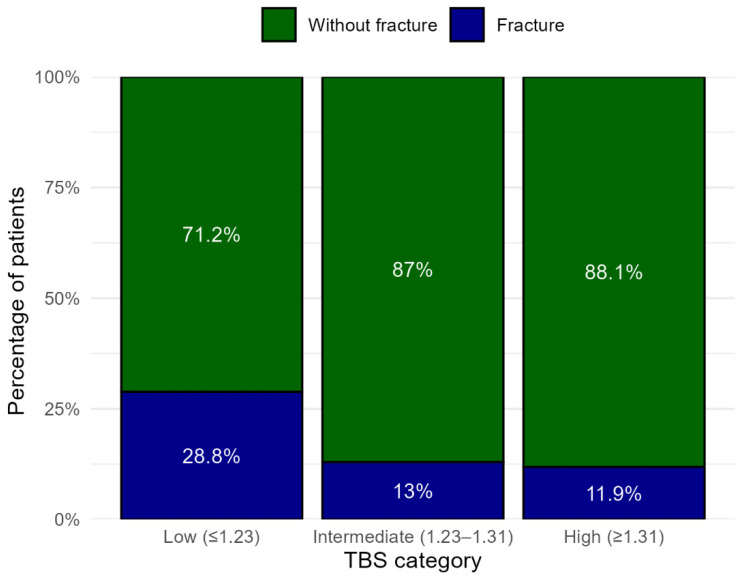
Percentage of patients with fractures and without fractures in our cohort, depending on the TBS category. The patients were categorized into low (≤1.23), intermediate (1.23–1.31), and normal TBS categories (≥1.31)—discriminative values of the TBS thresholds based on cutoff values proposed by McCloskey et al. and Omichi et al. [18,21].

To evaluate the discriminative value of the TBS thresholds proposed by McCloskey et al. (2015) and confirmed by Omichi et al. (2024), patients were categorized into degraded (≤1.23), partially degraded (1.23–1.31), and normal (≥1.31) TBS groups [18,21]. The prevalence of osteoporotic fractures was highest in the degraded (low) TBS group (28.8%) and significantly lower in the partially degraded (intermediate) (13.0%) and normal (high) (11.9%) categories (χ^2^ = 24.0, *p* < 0.001) (Figure 4). In logistic regression, low TBS was associated with a more than threefold higher risk of fracture compared to high TBS (OR = 3.0; 95% CI: 1.84–4.98; *p* < 0.001), whereas the intermediate group did not differ significantly (OR ≈ 1.1; *p* = 0.73).

Univariable logistic regression models were evaluated for fracture risk using TBS- and BMD-derived T-scores as predictors (Table 4).

**Figure 5:**
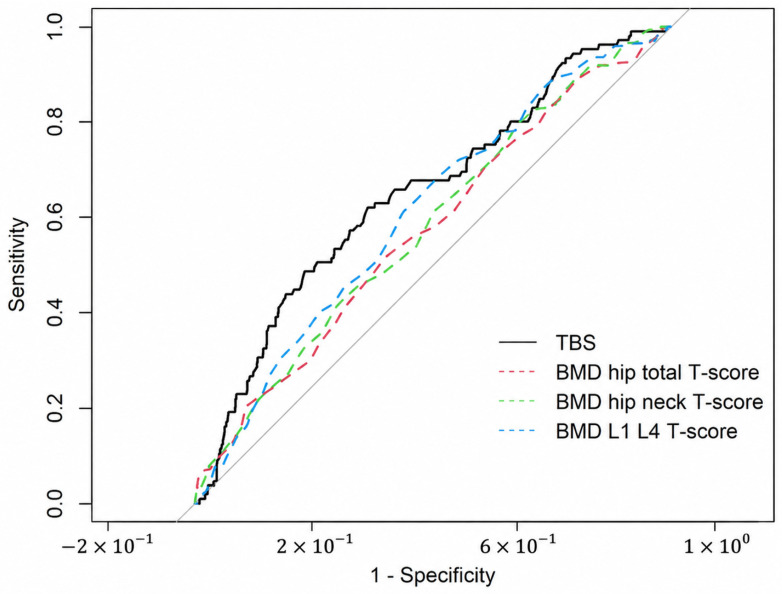
Receiver operating characteristic (ROC) curves for univariable models based on TBS, total hip T-score, femoral neck T-score, and L1–L4 T-score.

Univariable logistic regression models (Table 4) showed that lower TBS and BMD T-scores were significantly associated with higher fracture risk. For TBS T-score (Model 1, N = 596), each 1 SD decrease increased fracture risk by 56% (OR = 1.56, 95% CI 1.26–1.94, *p* < 0.001), while TBS (Model 2, N = 634) conferred a 67% higher risk per 1 SD decrease (OR = 1.67, 95% CI 1.34–2.08, *p* < 0.001). BMD-based models also showed significant associations: hip total T-score (OR = 1.33), hip neck T-score (OR = 1.40), and L1–L4 T-score (OR = 1.47), all *p* < 0.001. Models based on TBS had lower AIC values, suggesting better model fit, despite smaller sample sizes. AUC analyses indicated superior discriminative ability of TBS (AUC = 0.649) compared with BMD hip total T-score (AUC = 0.573, *p* = 0.047, DeLong’s test) (Figure 5).

In the next step, we compared fracture accordance using single-variable BMD models versus multivariable models including TBS. Across all sites (hip total, hip neck, L1–L4), addition of TBS improved model fit (lower AIC) and discrimination (higher AUC). DeLong’s test confirmed that the multivariable models had significantly higher AUC than single-variable BMD models (*p* = 0.006–0.027). In the multivariable models, TBS remained a significant predictor, while the effect of BMD T-score was attenuated (Table 5).

Both absolute TBS values and their T-scores were significantly lower in women with fractures. The observed differences were highly statistically significant (*p* < 0.001), which strengthens the reliability of the findings.

The negative correlations between TBS and age, as well as between TBS and BMI, were observed. The strength of the correlation with age was markedly greater than with BMI (r = −0.36 to −0.38 vs. r = −0.14).

Application of the TBS thresholds proposed by McCloskey et al. in 2016 [18] and Omichi et al. in 2024 [21] allowed effective stratification of fracture risk. The highest fracture rate was observed in the low TBS group (28.8%), whereas the intermediate and high TBS groups showed significantly lower risk. Logistic regression analysis demonstrated that low TBS was associated with a more than threefold increase in fracture risk compared with high TBS (OR = 3.0; 95% CI: 1.84–4.98; *p* < 0.001). The lack of a significant difference in the intermediate group (OR ≈ 1.1; *p* = 0.73) may be due to heterogeneity within this group or to limited sample size, warranting further investigation.

In univariable logistic regression, lower TBS and DXA T-scores were significantly associated with higher fracture risk. TBS models showed lower AIC and higher AUC compared with DXA alone.

The addition of TBS to DXA-based models improved model fit and discrimination (AIC ↓, AUC ↑), with TBS remaining a significant predictor in multivariable models. TBS offers superior diagnostic accuracy compared to BMD alone, without reservations.

## 5. Discussion

The main value of our study is the confirmation, based on a large, representative single-centre cohort of Polish women aged 40 to 76 years, that extending DXA by adding TBS better correlates with fracture presence than BMD alone. The fracture incidence increases with decreasing TBS. This suggests their potential as independent markers of bone microarchitecture. The results are consistent with previous reports, indicating a deterioration of trabecular bone structure with advancing age and higher body mass. Moreover, TBS decreases significantly with age, and this relationship is more clinically relevant than the decline in BMD measured by DXA, with implications for fracture prevention in the elderly, independent of traditional BMD assessment.

In the study group, a low TBS was associated with a more than 3-fold increase in fracture risk, compared with a high TBS. Although the TBS cut-off values used in our analysis (≤1.23; 1.23–1.31 and ≥1.31) were not developed specifically for the Polish cohort, they are derived from previously published and widely used international reference categories for interpreting the TBS score [18]. Although these values have demonstrated clinical utility in external cohorts and are supported by previous publications and expert guidelines, their direct application to the Polish population should be interpreted with caution, and requires future prospective validation in a dedicated Polish cohort.

In the entire population, both BMD and TBS distinguished between patients with fractures and those without fractures. In subgroup analyses among women with low bone mass and osteoporosis, only TBS showed a statistically significant difference between patients with fractures and those without fractures, whereas BMD did not. These results suggest that TBS may provide additional clinical information for assessing fracture risk in women with low bone mass or osteoporosis; however, interpretation should be undertaken with caution, given the sample size in the subgroups and the resulting limited statistical power. The main contribution of this study is, therefore, not to establish TBS as a new independent prognostic factor, but to provide one of the largest Polish datasets confirming the value of TBS in routine fracture risk stratification and to confirm its utility in a Central European cohort.

Osteoporosis is the major cause of fractures. In Western populations, both the number of hip fractures and the number of hip prostheses have been rising in recent decades [22,23]. This is the first clinic-based cohort study in Poland conducted on a homogeneous, large group at risk for osteoporosis development—women over 40 years of age. The results are consistent with numerous similar studies conducted worldwide.

Kanis et al. [24], in the European guidance for the diagnosis and management of osteoporosis in postmenopausal women, emphasize that TBS may complement the FRAX calculator in estimating the 10-year fracture risk. An extended version of FRAX incorporating TBS is available online (https://www.sheffield.ac.uk/TBS/CalculationTool.aspx, accessed on 27 January 2026). Moreover, TBS has been included as a highly important prognostic factor in the most recent FRAX Plus fracture risk calculator, available at https://www.fraxplus.org/ (accessed on 27 January 2026) [25,26]. In addition, TBS itself is an independent risk factor for fractures.

Years of research have demonstrated that BMD values differ substantially across races and ethnic groups [27]. Consequently, the risk of osteoporotic fractures also varies considerably between populations. Global studies have identified up to a tenfold difference in fracture risk across regions [28]. For this reason, more than 45 versions of the FRAX fracture-risk calculator have been developed for 63 different countries, ethnic groups, and nationalities [20].

The largest and best-known studies confirming the association between reduced TBS and a higher risk of osteoporotic fractures come from Canadian populations. These were extensive cohort studies evaluating data from the Manitoba Bone Density Program registry, conducted in the province of Manitoba, Canada. This registry is not a single study, but an ongoing monitoring program of DXA scans since 1990, operating as an integrated program since 1997 to the present day. Various analyses covering different cohorts and observation periods have been produced, including the 2011 study by Hans D. et al. (Manitoba study), which assessed TBS in over 29,000 women [29], and the 2014 study by Leslie WD. et al., presenting TBS results from over 3000 men aged ≥ 50 [30]. Overall, TBS was assessed in 73,108 individuals aged 40 or older, of whom 90% were women; the mean age was 64 years, and the mean observation period was 4.7 years. The study showed that TBS complements BMD in predicting major osteoporotic fractures (MOFs), hip fractures (HFs), and all fractures related to osteoporosis, independently of BMD. Decreases in BMD values and TBS complement each other in improving fracture risk prediction in persons over 40 years of age [31,32]. Interestingly, no such relationship was found in people under 40 years of age. It seems that TBS has not confirmed clinical significance in this younger patient group [33]. For this reason, women aged 40 years and older were selected for our study.

Several studies have addressed Asian populations; for example, a prospective, 10-year Japanese Population-based Osteoporosis (JPOS) study confirmed that TBS was an independent predictor of vertebral fractures in Japanese women aged 50 years or older. Furthermore, the combined assessment of TBS and BMD improved fracture risk estimation in this population [34]. Another prospective study from Hong Kong in elderly individuals (mean age 73 years) demonstrated that adding TBS calculation to BMD assessment improves the prediction of major osteoporotic fractures (MOFs) more reliably than BMD alone. However, the authors suggest considering separate, validated thresholds for different populations [35].

A study from Thailand supports the role of additional TBS assessment in postmenopausal women with and without fractures, in which the fracture group showed significantly lower TBS, and a 1 SD reduction was associated with a higher fracture risk ratio (OR) [36]. Numerous studies have established reference norms and evaluated the clinical value of TBS in various populations, including those with comorbidities associated with secondary osteoporosis such as type 2 diabetes mellitus (T2DM), hypercortisolism, chronic kidney disease, glucocorticoid-induced osteoporosis (GIO) and rheumatological conditions: rheumatoid arthritis, ankylosing spondylitis, polymyalgia rheumatica, and systemic lupus erythematosus [7]. These studies also highlight ethnic differences in TBS values, and advocate the use of population-specific thresholds [21].

Studies conducted in Europe, including the FRODOS cohort from Spain [37] and the NoFRACT study from Norway [38], confirmed that patients with fractures had significantly lower TBS than individuals without fractures. It is worth noting the study conducted in France in 2013 (OFELY study), which highlighted the fact that TBS improves fracture risk prediction in a population of non-osteoporotic women, and enables the identification of individuals at risk of fracture despite relatively normal BMD values [39].

The multi-centre Eastern European Study included 1031 women aged 45 years and older from Serbia, Bulgaria, Romania, and Ukraine. Investigators evaluated the role of TBS in a routine clinical practice as a complement to BMD. They found that combining BMD and TBS improved sensitivity by 28%, accuracy by 17%, and specificity by 9%, in fracture prediction [3]. Despite the large number of studies worldwide on the application of TBS in postmenopausal osteoporosis, only two studies have been conducted in Poland: a pilot study in 2018 (35 women), [9] and a group of 102 women, where differences observed between TBS, BMD, and handgrip strength regarding osteoporotic fractures were found to be not statistically significant [10]. The small sample sizes reduced the statistical power of these studies. To our knowledge, our project is the largest study on TBS in the Polish population.

The final results of our study confirm the significant diagnostic value of TBS in assessing the association with fracture occurrence.

The diagnostic value of BMD alone is insufficient for assessing fracture risk. TBS may serve as a valuable complement to added conventional BMD measurement, particularly when BMD does not reflect actual fracture risk. Future directions should consider prospective studies to evaluate the long-term predictive value of TBS. Additionally, integrating TBS with other clinical indicators and analysing its treatment response variability could substantially enrich osteoporosis diagnostics, and should be implemented in routine clinical practice.

Strengths of this study include a relatively large and reasonably homogeneous cohort that reflects the real population risk. The patients did not use osteoporosis medications that could have affected the study results.

Limitations of the study include its cross-sectional design, the absence of data on fracture timing, location, and circumstances, and potential measurement bias in TBS among individuals with high BMI. Additionally, important fracture risk factors such as lifestyle, pharmacotherapy, substance use, and comorbidities were not accounted for.

The sample size was not very large, compared with the huge cohorts described in the literature, and the single-centre setting and exclusion of men further limit generalizability. Nonetheless, these constraints do not detract from the value of the findings, which contribute valuable insights into the predictive utility of bone microarchitecture assessment for fracture risk.

The findings of our study highlight the critical importance of incorporating TBS into routine clinical evaluation of bone strength in Polish women. Notably, TBS provides complementary information to that of bone mineral density (BMD), and improves fracture risk assessment when integrated with tools such as FRAX. Integrating TBS with other clinical risk factors significantly enhances the precision of osteoporosis diagnostics and patient management strategies.

Our results are consistent with the ESCEO/IOF expert recommendations [7], which position trabecular bone score (TBS) as a complementary tool that enhances fracture-risk assessment beyond BMD and FRAX, particularly in women with low bone mass or osteoporosis. TBS is a particularly valuable tool in the risk assessment process, especially for patients who are close to the threshold of requiring therapeutic intervention according to the FRAX probability score or a BMD T-score of approximately (−2.5) [7].

For example, patients with osteopenia account for the highest burden of fractures [40]. In these cases, adding TBS to BMD (combined T-score) and calculating the TBS-adjusted FRAX allows for a precise risk assessment. This enables selection of the optimal timing for initiating treatment, thereby avoiding delays or overtreatment. Incorporating TBS into predictive tools such as FRAX, can enhance prognostic accuracy and support therapeutic decision-making.

Future prospective studies should validate TBS thresholds in the Polish population, clarify its long-term predictive value, and assess responsiveness to therapeutic interventions. These studies should focus on TBS’s impact on long-term outcomes and influence on decision-making. Cost-effectiveness analyses combining TBS with BMD for osteoporosis diagnosis and treatment in Poland are also needed.

## 6. Conclusions

The results of this study are consistent with data from other countries, and indicate the usefulness of TBS assessment in diagnosing osteoporosis and evaluating fracture risk. They confirm and emphasise the importance of incorporating TBS into the routine assessment of skeletal strength in Polish women, thereby facilitating decisions regarding the initiation of antiresorptive or anabolic treatment.

## Figures and Tables

**Table 1:** Patient characteristics.

	All (*N* = 933) (100%)	Without Fracture (*N* = 760) (81.46%)	With Fracture (*N* = 173) (18.54%)	*p*-Value
Bone mineral density				
Normal BMD	281 (30.12%)	248 (32.63%)	33 (19.08%)	0.001 *
Osteopenia	457 (48.98%)	369 (48.55%)	88 (50.87%)	
Osteoporosis	195 (20.90%)	143 (18.82%)	52 (30.06%)	
Age				
mean ± sd	60.02 ± 5.89	59.82 ± 5.93	60.91 ± 5.64	0.02 **
BMI				
mean ± sd	26.18 ± 4.20	26.10 ± 4.22	26.53 ± 4.09	ns **
BMD hip total T-score				
N; mean ± sd	918; −0.64 ± 1.00	747; −0.59 ± 0.99	171; −0.87 ± 1.02	0.001 **
BMD neck T-score				
N; mean ± sd	926; −1.04 ± 0.98	753; −0.98 ± 0.98	−1.29 ± 0.90	<0.001 **
BMD L1–L4 T-score				
N; mean ± sd	921; −1.14 ± 1.34	749; −1.05 ± 1.35	172; −1.52 ± 1.21	<0.001 **
BMD L1–L4				
N; mean ± sd	635; 0.92 ± 0.14	527; 0.93 ± 0.14	108; 0.88 ± 0.13	0.002 **
TBS T-score				
N; mean ± sd	596; −1.98 ± 1.17	492; −1.89 ± 1.15	104; −2.40 ± 1.16	<0.001 **
TBS				
N; mean ± sd	634; 1.29 ± 0.11	529; 1.30 ± 0.10	105; 1.25 ± 0.10	<0.001 **

*N*—number of patients in cases with missing data; mean ± SD—mean ± standard deviation; *p*-value—value for the difference between patients with and without fracture; ns—not statistically significant; * Pearson’s chi-squared test; ** Welch two-sample *t*-test.

**Table 2:** FRAX-based fracture risk estimates (with and without BMD and TBS adjustment).

FRAX	Without Fracture N = 760 No. obs. ^1^/Mean (SD)/Median (Q1, Q3)	Fracture N = 173 ^1^ No. obs./Mean (SD)/Median (Q1, Q3)
FRAX MOF	731/5.91 (2.24)/5.70 (4.30, 7.20)	168/8.56 (3.83)/7.80 (5.75, 10.00)
FRAX BMD MOF	727/5.13 (2.25)/4.90 (3.40, 6.40)	170/7.52 (4.77)/6.25 (4.80, 8.80)
TBS adjusted FRAX BMD MOF	497/5.61 (2.69)/5.06 (3.77, 6.88)	103/8.71 (5.82)/7.24 (5.41, 10.24)
FRAX HF	729/1.32 (1.02)/1.00 (0.60, 1.70)	168/2.43 (1.84)/2.00 (1.20, 3.05)
FRAX BMD HF	727/0.83 (0.98)/0.50 (0.20, 1.10)	170/1.71 (2.23)/1.00 (0.40, 2.20)
TBS adjusted FRAX BMD HF	492/0.92 (1.12)/0.53 (0.22, 1.13)	103/2.09 (2.80)/1.19 (0.50, 2.65)

^1^ Number of patients in cases with missing data, MOF—major osteoporotic fractures; HF—hip fracture).

**Table 3:** Pearson correlations of TBS and TBS T-score with age and BMI.

Predictor	Outcome	Pearson r	*p*	N
BMI	TBS BMD	−0.140	<0.001	634
BMI	TBS T-score	−0.142	<0.001	596
Age	TBS T-score	−0.361	<0.001	596
Age	TBS BMD	−0.382	<0.001	634

Pearson correlation coefficients (r), *p*-values, and sample sizes (N) are reported for each pair of variables.

**Table 4:** Logistic regression models.

Model	Predictor	OR (95%CI)	AIC	*p* Value	N
Model1	TBS T-score	1.56 (1.26, 1.94)	539.06	<0.001	596
Model2	TBS BMD	1.67 (1.34 2.08)	550.89	<0.001	634
Model3	DXA hip total T-score	1.33 (1.13, 1.59)	875.41	<0.001	918
Model4	DXA hip neck T-score	1.40 (1.18, 1.68)	881.06	<0.001	926
Model5	DXA L1 L4 T-score	1.47 (1.23, 1.77)	872.59	<0.001	921

OR: Odds ratio for the occurrence of fractures in patients with TBS and DEXA values lower by 1 SD. N: Number of observations included in the model. AIC: Akaike Information Criterion.

**Table 5:** Univariable and multivariable logistic regression models for fracture risk prediction.

DXA Site	Model	Predictor	OR (1 SD Decrease)	95% CI	*p*	N	AIC	AUC
hip total	Univariable	DXA T-score	1.439	1.22–1.70	**	624	559.6	0.585
hip total	Multivariable	DXA T-score	1.247	0.98–1.58	+	624	545.5	0.656
		TBS BMD	1.587	1.36–1.85	***			
hip neck	Univariable	DXA T-score	1.558	1.30–1.87	***	628	557.4	0.606
hip neck	Multivariable	DXA T-score	1.337	1.02–1.75	*	628	545.4	0.655
		TBS BMD	1.547	1.32–1.81	***			
L1–L4	Univariable	DXA T-score	1.513	1.29–1.77	***	627	555.4	0.608
L1–L4	Multivariable	DXA T-score	1.193	0.87–1.63	ns	627	545.0	0.656
		TBS BMD	1.556	1.33–1.82	***			

OR = odds ratio per 1 SD decrease; AUC = area under the ROC curve; N = number of observations included in model; AIC = Akaike Information Criterion; *p*-value notation: *** *p* < 0.001, ** *p* < 0.01, * *p* < 0.05, + *p* < 0.1, ns = not significant.

## Data Availability

Some or all of the framework features that support the findings of this study are available from the corresponding author upon reasonable request.

## Supplementary Materials

The following supporting information can be downloaded at: https://www.mdpi.com/article/10.3390/jcm15114185/s1, Table S1. Characteristics of patients with osteoporosis; Table S2. Characteristics of patients with osteopenia. Table S3. Characteristics of patients with normal BMD results. Table S4. ROC Analysis Summary.

## Abbreviations

TBS	Trabecular bone score
DXA	Dual-energy X-ray absorptiometry
WHO	World Health Organisation
BMD	Bone mineral density
SD	Standard deviations
NICE	National Institute for Health and Care Excellence
FRAX	Fracture Risk Assessment Tool
IOF	International Osteoporosis Foundation
ESCEO	European Society for Clinical and Economic Aspects of Osteoporosis, Osteoarthritis and Musculoskeletal Diseases
ISCD	International Society for Clinical Densitometry
BMI	Body Mass Index
OR	Odds Ratio
CI	Confidence intervals
AUC	Area under the curve
AIC	Akaike Information Criterion
BIC	Bayesian Information Criterion
N	Number of patients
*p*-value	Value of the difference
MOF	Major osteoporotic fractures
T2DM	Type 2 diabetes mellitus
GIO	Glucocorticoid-induced osteoporosis
