# Supplementary material for: The Added Value of Trabecular Bone Score in Evaluating Fracture Risk Among Polish Women Aged 40–76 Years
Source: J Clin Med. 2026 May 28;15(11):4185. doi: 10.3390/jcm15114185 (PMC13258481; doi:10.3390/jcm15114185)
Supplement: Supplementary file 1 [file jcm-15-04185-s001.zip › jcm-4286532-supplementary.pdf]

Table S1

Characteristics of patients with osteoporosis

	All (N = 195)	Without fracture (N = 143)	With fracture (N = 52)	Wartość P
**Age**				
mean ± sd	61.69 ± 5.45	61.81 ± 5.55	61.37 ± 5.18	ns*
**BMI**				
mean ± sd	24.54 ± 3.80	24.48 ± 3.95	24.69 ± 3.40	ns**
**BMD hip total T-score**				
N; mean ± sd	191; -1.62 ± 0.75	139; -1.59 ± 0.67	-1.72 ± 0.92	ns**
**BMD neck T-score**				
N; mean ± sd	193; -1.95 ± 0.76	141; -1.90 ± 0.78	-2.09 ± 0.69	ns*
**BMD L1-L4 T score**				
N; mean ± sd	194; -2.68 ± 0.76	142; -2.66 ± 0.81	-2.73 ± 0.58	ns*
**BMD L1-L4**				
N; mean ± sd	121; 0.76 ± 0.09	94; 0.76 ± 0.09	27; 0.75 ± 0.08	ns*
**TBS T score**				
N; mean ± sd	117; -2.90 ± 1.00	91; -2.81 ± 1.02	26; -3.25 ± 0.85	**0.029****
**TBS**				
N; mean ± sd	120; 1.22 ± 0.11	93; 1.23 ± 0.12	27; 1.18 ± 0.08	**0.045***

N – number of patients in cases with missing data; mean ± SD – mean ± standard deviation; P-value – value for the difference between patients with and without fracture; ns – not statistically significant; * Pearson's chi-squared test; ** Welch two-sample t-test.

Table S2

Characteristics of patients with osteopenia

	All (N = 457)	Without fracture (N = 369)	With fracture (N = 88)	Wartość P
**Age**				
mean ± sd	60.42 ± 5.65	60.23 ± 5.62	61.20 ± 5.73	ns*
**BMI**				
mean ± sd	26.02 ± 4.06	25.92 ± 4.14	26.48 ± 3.67	ns**
**BMD hip total T-score**				
N; mean ± sd	449; -0.78 ± 0.74	363; -0.78 ± 0.75	86; -0.78 ± 0.70	ns**
**BMD neck T-score**				
N; mean ± sd	454; -1.22 ± 0.68	366; -1.22 ± 0.69	-1.23 ± 0.64	ns*

**BMD L1-L4 T score**				
N; mean ± sd	448; -1.31 ± 0.79	361; -1.29 ± 0.80	87; -1.39 ± 0.74	ns*
**BMD L1-L4**				
N; mean ± sd	324; 0.90 ± 0.09	259; 0.91 ± 0.09	65; 0.89 ± 0.09	ns*
**TBS T score**				
N; mean ± sd	303; -2.04 ± 1.04	241; -1.96 ± 1.01	62; -2.34 ± 1.07	**0.013****
**TBS**				
N; mean ± sd	323; 1.29 ± 0.09	261; 1.30 ± 0.09	62; 1.26 ± 0.09	**0.004****

N *– number of patients in cases with missing data;* ***mean ± SD*** *– mean ± standard deviation;* ***P-value*** *– value for the difference between patients with and without fracture;* ***ns*** *– not statistically significant; * Pearson's chi-squared test; ** Welch two-sample t-test.*

Table S3

Characteristics of patients with normal BMD results

	All (N = 281)	Without fracture (N = 248)	With fracture (N = 33)	Wartość P
**Age**				
mean ± sd	58.21 ± 6.12	58.06 ± 6.13	59.39 ± 6.01	ns*
**BMI**				
mean ± sd	27.58 ± 4.24	27.31 ± 4.15	29.60 ± 4.40	0.007**
**BMD hip total T-score**				
N; mean ± sd	278; 0.26 ± 0.71	245; 0.26 ± 0.72	0.23 ± 0.64	ns*
**BMD neck T-score**				
N; mean ± sd	279; -0.11 ± 0.72	246; -0.09 ± 0.75	-0.20 ± 0.50	ns*
**BMD L1-L4 T score**				
N; mean ± sd	279; 0.20 ± 0.99	246; 0.23 ± 1.00	0.04 ± 0.94	ns*
**BMD L1-L4**				
N; mean ± sd	190; 1.06 ± 0.10	174; 1.06 ± 0.10	16; 1.06 ± 0.12	ns*
**TBS T score**				
N; mean ± sd	176; -1.25 ± 1.00	160; -1.25 ± 1.02	16; -1.25 ± 0.82	ns**
**TBS**				

N; mean ± sd	191; 1.35 ± 0.09	175; 1.35 ± 0.09	16; 1.34 ± 0.09	ns*

***N** – number of patients in cases with missing data; **mean ± SD** – mean ± standard deviation; **P-value** – value for the difference between patients with and without fracture; **ns** – not statistically significant; * Pearson's chi-squared test; ** Welch two-sample t-test.*

Table S4

ROC Analysis Summary

Model	Optimal cut-off	Sensitivity	Specificity	PPV	NPV
BMD hip total T-score	0.192	0.520	0.594	0.227	0.844
BMD hip neck T-score	0.158	0.798	0.321	0.213	0.874
BMD L1-L4 T-score	0.189	0.610	0.563	0.243	0.863
TBS	0.168	0.619	0.637	0.253	0.894

Optimal probability cut-off values were determined using the Youden index.
